# MicroRNA Expression Profiling of Lactating Mammary Gland in Divergent Phenotype Swine Breeds

**DOI:** 10.3390/ijms16011448

**Published:** 2015-01-08

**Authors:** Jing Peng, Jun-Sheng Zhao, Yi-Fei Shen, Hai-Guang Mao, Ning-Ying Xu

**Affiliations:** College of Animal Sciences, Zhejiang University, Hangzhou 310058, China; E-Mails: pennypeng1987@163.com (J.P.); zjs4939@126.com (J.-S.Z.); 06dwkxsyf@zju.edu.cn (Y.-F.S.); maohaiguang@163.com (H.-G.M.)

**Keywords:** microRNA sequencing, mammary gland, swine

## Abstract

MicroRNA (miRNA) plays a key role in development and specific biological processes, such as cell proliferation, differentiation, and apoptosis. Extensive studies of mammary miRNAs have been performed in different species and tissues. However, little is known about porcine mammary gland miRNAs. In this study, we report the identification and characterization of miRNAs in the lactating mammary gland in two distinct pig breeds, Jinhua and Yorkshire. Many miRNAs were detected as significantly differentially expressed between the two libraries. Among the differentially expressed miRNAs, many are known to be related to mammary gland development and lactation by interacting with putative target genes in previous studies. These findings suggest that miRNA expression patterns may contribute significantly to target mRNA regulation and influence mammary gland development and peak lactation performance. The data we obtained provide useful information about the roles of miRNAs in the biological processes of lactation and the mechanisms of target gene expression and regulation.

## 1. Introduction

MicroRNA (miRNA) belongs to a class of evolutionary conserved small RNA (19–25 nucleotides) that regulates gene expression in a sequence-specific manner, essentially at the posttranscriptional level. miRNAs regulate target genes through either the degradation of messenger RNA (mRNA) or via translational inhibition [1]. miRNA plays a key role in development and specific biological processes, such as cell proliferation, differentiation, and apoptosis [2]. The mammary gland, unlike most organs, undergoes the majority of its growth and development postnatally, with maximal growth and development following the onset of pregnancy and during early lactation [3]. The complex initiation of mammary gland lactation has been extensively studied over the years at the genetic, physiological and morphological levels because of its important functions [4]. It has been reported that many genes are expressed differently to maintain lactation [5]. During lactation, sow milk production is one of the most important factors limiting neonatal piglet growth and survival. So milk yield in sows is a critical factor for economic success of swine operations because it is the main determinant of litter growth [6].

Extensive studies of mammary miRNAs have been performed in mouse [7,8,9], cow [5,10,11], goats [12], sheep [3], and human [4,13]. These studies provide insight into the types of miRNAs and their possible mechanisms in regulating mammary gland development and lactation for these species. However, little is known about porcine mammary gland miRNAs. Notably, there have been no reports on miRNA expression in the porcine mammary gland in different pig breeds during the peak lactation to date.

Therefore, in this study, we report the identification and characterization of miRNAs in the mammary gland in two distinct pig breeds. Two miRNA libraries were constructed from mammary gland samples taken from Jinhua (RX_J) and Yorkshire (RX_Y) at 21-day lactation period. Jinhua and Yorkshire are two distinct pig breeds in many ways, including lactation characteristics which make them ideal experimental models for studying mammary gland development and regulation of lactation. The comparison of expression levels of miRNAs in RX_J and RX_Y were analyzed and many miRNAs were detected as significantly differentially expressed between two libraries. Target gene analysis between the two libraries also illustrated that miRNA could be involved in regulating gene function during lactation. The data we obtained provide useful information about the roles of miRNAs in the biological processes of lactation and the mechanisms of target gene expression and regulation.

## 2. Results

### 2.1. Determination of Porcine Mammary Gland

**Figure 1:**
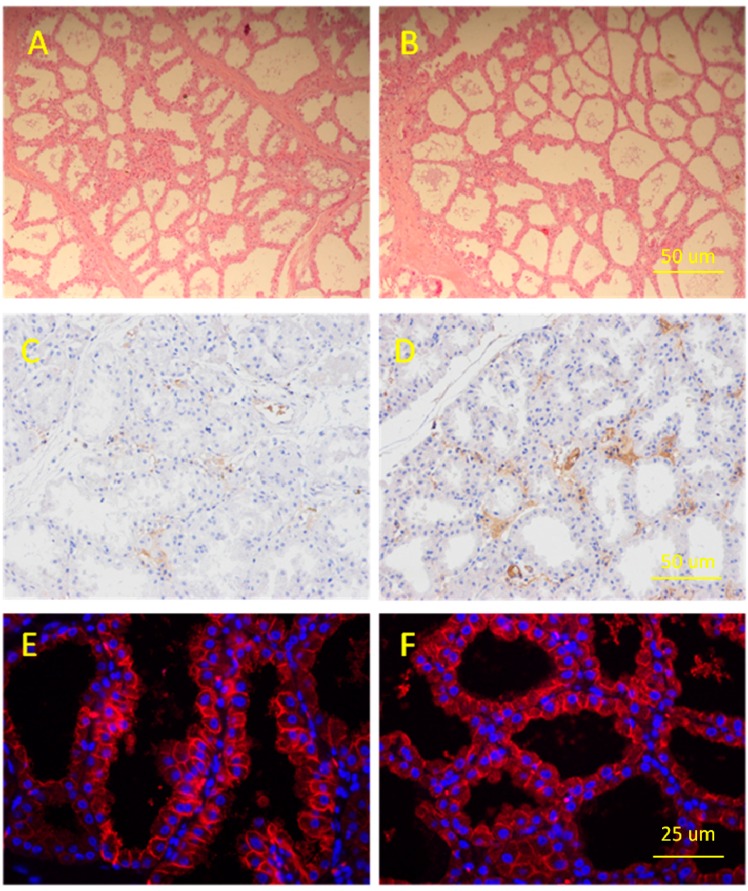
The microstructure and gene expression of lactating mammary gland tissue in Jinhua and Yorkshire pig breeds. (**A**) Paraffin section of Jinhua mammary gland in the 21-day lactation period; (**B**) Paraffin section of Yorkshire mammary gland in the 21-day lactation period; (**C**) Expression of casein in Jinhua lactating mammary gland; (**D**) Expression of casein in Yorkshire lactating mammary glands; (**E**) Expression of cytokeratin in lactating Jinhua mammary glands and (**F**) Expression of cytokeratin in lactating Yorkshire mammary glands.

Hematoxylin and eosin (HE) staining, immunohistochemistry and immunofluorescence were all employed to verify the microstructure of the lactating mammary gland tissues used in constructing miRNA libraries. In the lactation mammary glands, many mature alveolar structures were packed with mammary lobules of a variety of shapes. Mature alveolar lumens seemed larger in Yorkshire than Jinhua in appearance and many of the lumens in both breeds were filled with secretions, with little connective tissue between alveoli (Figure 1A,B), and certain amounts of α-casein were found surrounding the nuclei and in the large alveolus. Additionally, α-casein expression level in Jinhua was comparatively lower than Yorkshire (Figure 1C,D). Furthermore, expression of cytokeratin 18 (CK-18), a marker of epithelial cells, was also detected in both mammary gland tissues (Figure 1E,F).

### 2.2. Analysis of Sequencing Data

**Figure 2:**
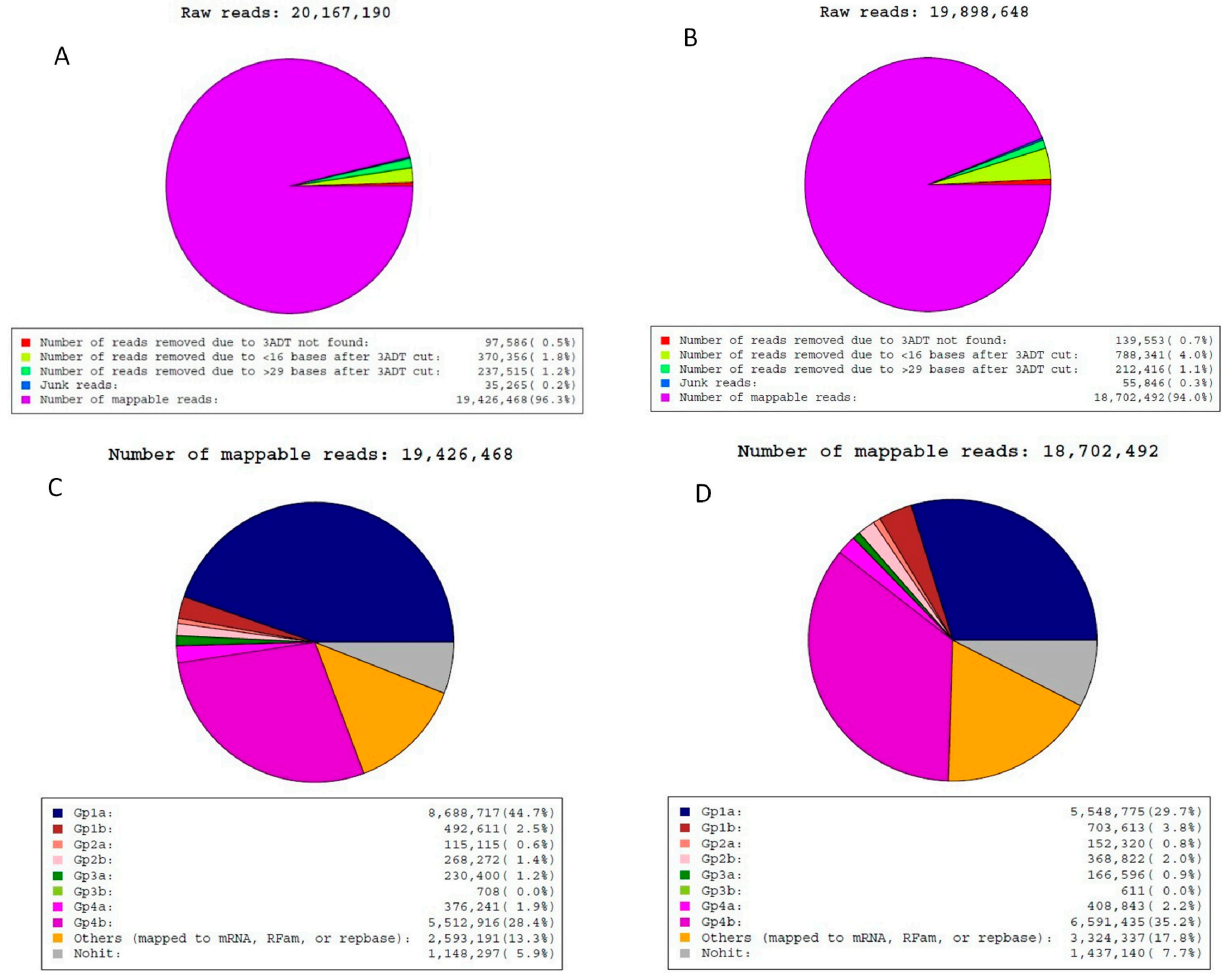
Reads obtained from RX_J and RX_Y libraries. Raw reads obtained from RX_J library (**A**) and RX_Y library (**B**). Mappable reads obtained from RX_J library (**C**) and RX_Y library (**D**). Group 1: Reads mapped to porcine miRNAs in miRbase and mapped to porcine genome; Group 2: Reads unmapped to porcine miRNAs in miRbase but mapped to porcine genome; Group 3: Reads unmapped to porcine genome; Group 4: Reads unmapped to porcine mammalian miRNAs in miRbase.

Two miRNA libraries were constructed using small RNA isolated from porcine mammary glands and sequenced using an Illumina Hiseq2500. A data analysis flowchart of the study is shown in Figure S1. A total of 20,167,190 raw reads from the RX_J library and 19,898,648 raw reads from the RX_Y library were obtained (Figure 2A,B). The ratio of RX_J/RX_Y was 101.3%, indicating that the two libraries were well represented. After filtering low-quality reads and adaptor sequences, 19,426,468 and 18,702,492 mappable reads were obtained from the RX_J and RX_Y libraries, respectively (Figure 2C,D). Next, the small RNAs were classified into different categories according to their annotations. After filtering reads mapped to the mRNA, RFam, or repbase, there were 8,688,717 and 5,548,775 reads representing 44.7% and 29.7% of the total mappable reads in RX_J and RX_Y libraries mapped to the porcine miRs in miRBase version 19.0, respectively.

**Figure 3:**
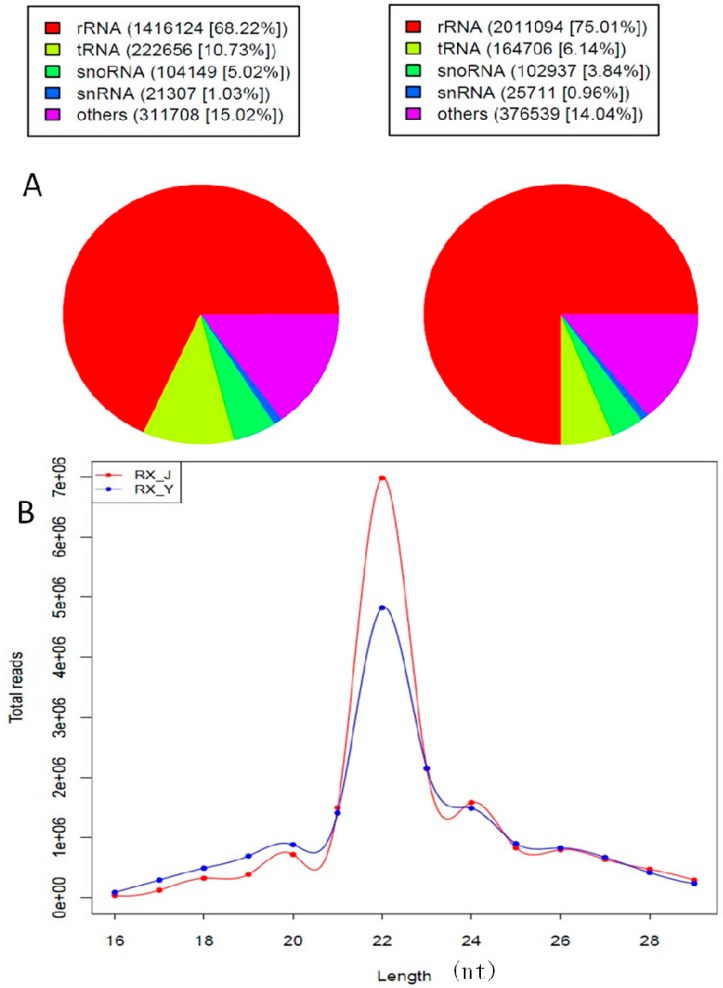
(**A**) Pie chart for Rfam RX_J and RX_Y and (**B**) Length distribution analysis.

We separated out and discarded rRNA, tRNA, snRNA, scRNA, srpRNA and snoRNA sequences, which were identified using a BLAST against the known noncoding RNAs deposited in the Rfam and NCBI GenBank databases (Figure 3A).

To assess the sequencing quality, we analyzed the length distribution based on both total abundance and distinct sequences. Length distribution analysis revealed that 41.51% and 31.38% were 22 nt in length for RX_J and RX_Y, consistent with the typical size range of miRNA (Figure 3B).

The unique miRNAs were categorized into three groups based on their hits: 406 miRNAs matched with known *ssc* miRNAs registered in the miRbase database; 135 and 138 miRNAs respectively were conserved among other mammals but have yet to be identified in porcines in Jinhua and Yorkshire library; 2823 and 2286 were predicted miRNAs. Predicted miRNAs were separated to three categories: Mapped to known miR of selected species and genome; Mapped to known miRs and miRs of selected species but unmapped to genome; Unmapped to known miRs but mapped to genome and within hairpins. A summary of different kinds of miRNAs is listed in Table 1.

**Table 1:** Known and predicted miRNAs.

Category	Jinhua Mammary Gland	Yorkshire Mammary Gland
Total miRNAs	3364	2830
Known miRNAs	406	406
Conserved miRNAs	135	138
Predicted miRNAs	2823	2286
Mapped to known miRs of selected species and genome	552	587
Mapped to known miRs and miRs of selected species but unmapped to genome	571	595
Unmapped to known miRs but mapped to genome and within hairpins	1700	1104

### 2.3. Comparison of Expression Levels of miRNAs in RX_J and RX_Y

**Figure 4:**
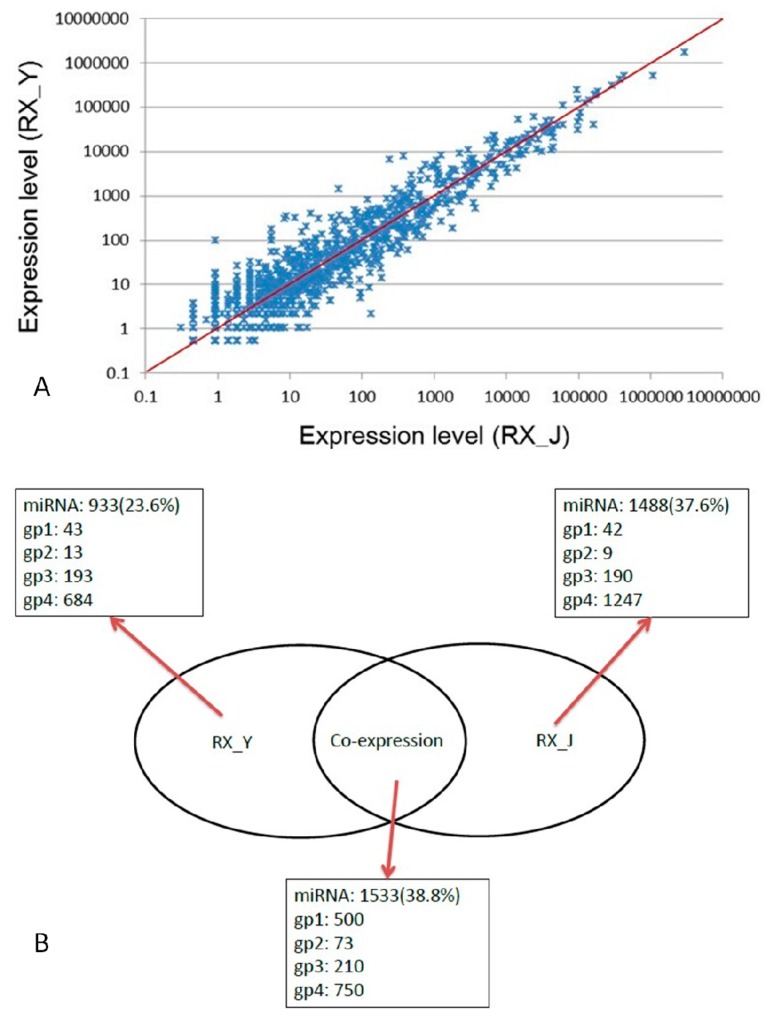
(**A**) Correlation of the two parallel libraries and (**B**) Expressed miRNA numbers in two libraries.

Correlation of the two parallel libraries was shown in Figure 4A. The X- and Y-axes show the expression levels of miRNAs in the two samples. The data from the two libraries are highly correlated, most scatter plots are focused on the axis, and most of the expression levels are equivalent. The Venn diagram (Figure 4B) displays the distribution of the unique miRNAs found in RX_J and RX_Y. There were 1533 miRNAs coexpressed in the two breeds while 1433 miRNAs were only expressed in RX_J and 933 were only expressed in RX_Y.

### 2.4. The Distribution of miRNA on the Porcine Genome

**Figure 5:**
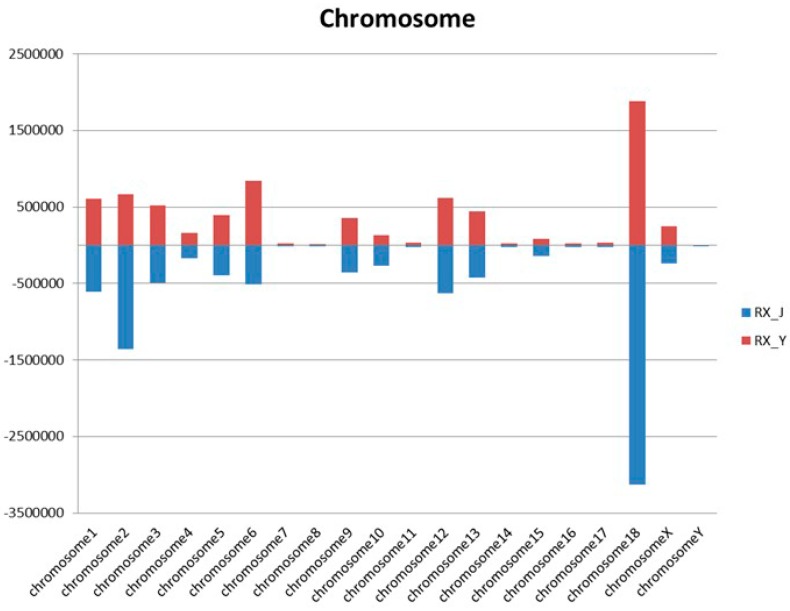
Chromosomal locations of miRNAs in porcine mammary glands.

We also analyzed the expression and distribution of the miRNAs in the porcine genome. Characteristics of chromosomal locations of miRNAs in porcine mammary glands were shown in Figure 5. We can see that chromosome 18 carries the most miRNAs in both pig breeds.

### 2.5. The Conservation Profile of Conserved Porcine miRNAs

**Figure 6:**
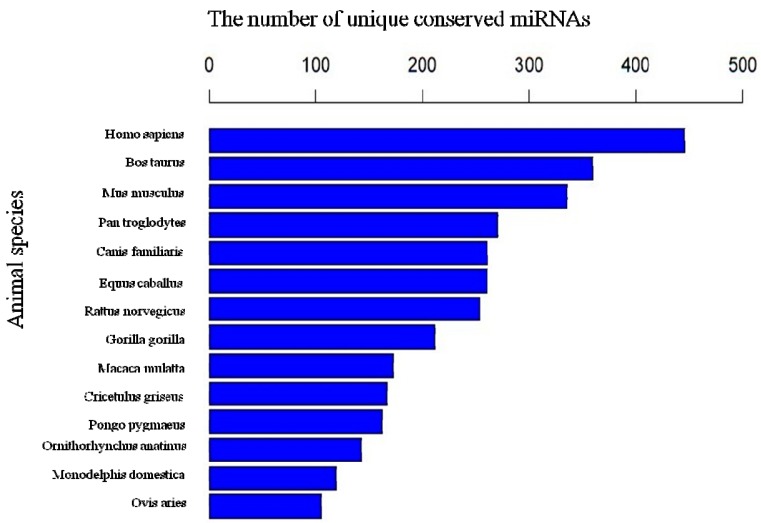
Conservation comparison of the identified miRNAs.

Conserved miRNA families are found in many animal species and have important functions in animal development and physiological processes. In our study, the sequences were also mapped to other mammalian genomes, and many miRNA families are conserved in a variety of animal species. For example, miR-542 was found in most species, including *Homo sapiens*, *Bos Taurus*, *Mus musculus*, *Pan troglodytes*, *Canis familiaris*, *Equus caballus*, *Rattus norvegicus*, *Gorilla gorilla*, and *Macaca mulatta*. The conservation of the identified miRNAs with other species is shown in Figure 6. In general, the porcine miRNA population is most conserved in *Homo sapiens*, *Bos taurus* and *Mus musculus.*

### 2.6. Identification of Differential Expression Patterns of miRNA in Porcine Mammary Gland

The identification of miRNAs that were differentially expressed between the two libraries was performed after their numbers were normalized to transcripts per million. The results show that a large number of miRNAs were differentially expressed (*p* < 0.05) between the two libraries, of which 46 known miRNAs were down-regulated and 166 were up-regulated in RX_Y compared to the RX_J (Table S1). Among the up-regulated miRNAs, ssc-miR-21 has the highest expression, while in the down-regulated miRNAs, ssc-let-7c has the highest expression level. The let-7 family miRNAs were all highly expressed in RX_J and RX_Y libraries. Some of the abundant miRNAs which were significantly differentially expressed (*p* < 0.01) between the two breeds are listed in Table 2.

**Table 2:** miRNAs that were significantly differentially expressed in the two libraries (Yorkshire relative to Jinhua).

miRNA Name	Sequence (5'–3')	Regulation	*p*-Value
ssc-miR-21	TAGCTTATCAGACTGATGTTGA	Up	0
ssc-miR-148b-3p	UCAGUGCAUCAGAACUUUGU	Up	0
ssc-miR-92a	TATTGCACTTGTCCCGGCCTGT	Up	3.4 × 10^−135^
ssc-miR-423-3p	AGCUCGGCUGAGGCCCCUCAGU	Up	1.1 × 10^−154^
ssc-miR-26	TTCAAGTAATCCAGGATAGGCT	Down	4 × 10^−155^
ssc-miR-24-3p	UGGCUCAGUUCAGCAGGAACAG	Down	1.37 × 10^−81^
ssc-miR-181a	AACAUUCAACGCUGUCGGUGAGUU	Down	1.67 × 10^−61^
ssc-miR-151-5p	UCGAGGAGCUCAGUCUAGU	Down	6.9 × 10^−4^

### 2.7. Gene Ontology (GO) Enrichment Analysis and Kyoto Encyclopedia of Genes and Genomes (KEGG) Pathway Analysis of Target Genes

**Figure 7:**
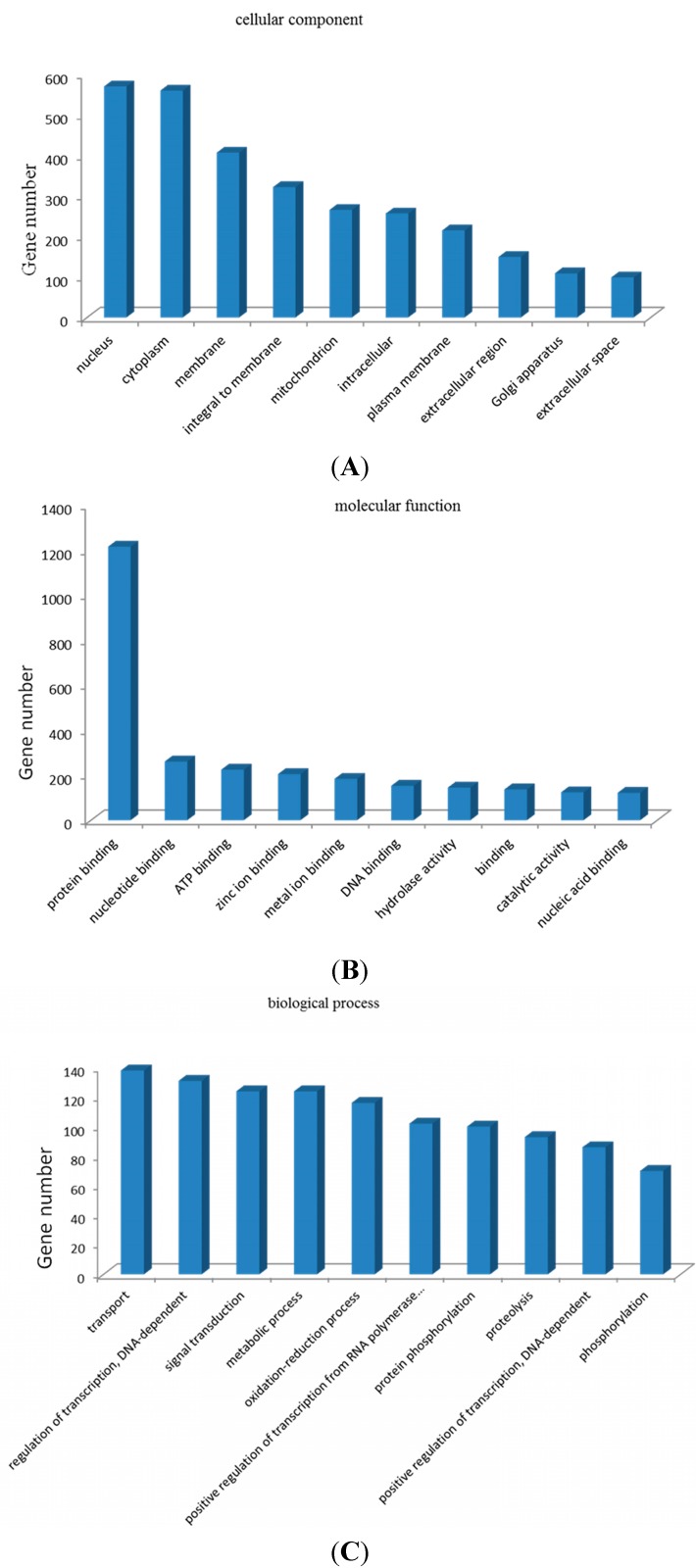
Partial GO enrichment for the predicted target genes in cellular component (**A**); molecular function (**B**) and biological processes (**C**).

Target gene prediction was performed to further understand the physiological functions and biological processes involving these miRNAs during mammary gland development and lactation, (Figure 7) based on miRNA/mRNA interactions to provide some molecular insight into the processes. The figure shows partial GO enrichment for the predicted target genes in cellular component, molecular function and biological processes. The enriched GO targets of the miRNAs were mainly associated with the nucleus, protein binding and transport items. The predicted target genes were classified according to KEGG function annotations to identify the pathways that were actively regulated by miRNAs in mammary gland (Tables S2 and S3). The involved pathways including the calcium signaling pathway, endocytosis, axon guidance, antigen processing and presentation, toll-like receptor signaling pathway, and the mammalian target of rapamycin (mTOR) signaling pathway may play important roles in the regulation of mammary gland differentiation and lactation.

### 2.8. Regulation of Lactation Genes by miRNAs

**Figure 8:**
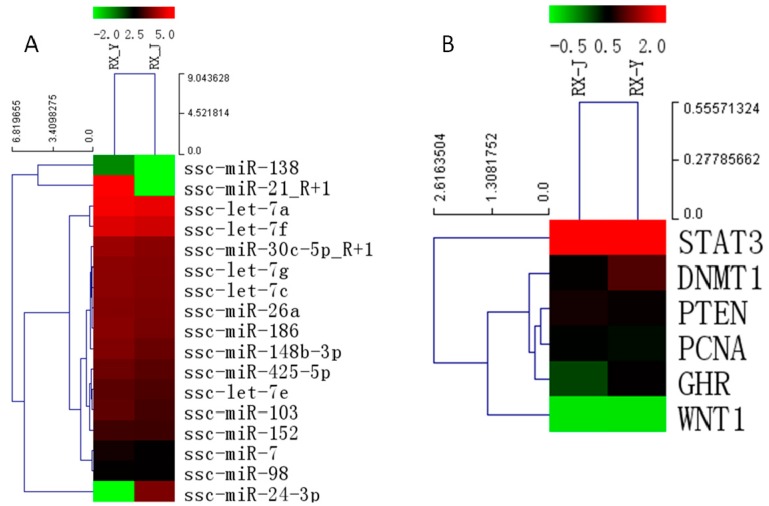
Hierarchical cluster analysis for lactation-related miRNAs (**A**) and target genes (**B**) (The color represent the expression level, green means low level red means high level; the number of the phylogenetic tree represents the evolutionary relationship of the genes, the smaller, the closer.)

Hierarchical cluster analysis for 17 lactation-related miRNAs expressed in mammary gland from Jinhua and Yorkshire lactation pigs was performed, and these miRNAs were found to be differentially expressed in the two libraries (Figure 8A). Some of these miRNAs were reported to be immune-related, like the miR-30 family, which can play a critical role in the development of the infant immune system [14]. Some miRNAs were shown to be related to mammary gland development and breast cancer. Therefore, we use Targetscan and Miranda to identify putative targets of these selected miRNAs. For example, DNMT1, PTEN and WNT1 were the putative target genes of miR-148/152 family. STAT3 is a target gene of miR-21 and PCNA is the target of miR-24-3p. Interestingly, many of these target genes are related to the proliferation and viability of mammary epithelial cells. GHR was demonstrated to play a key role in mammary gland development in many studies [15]. Thus, these related genes were then selected to perform hierarchical cluster analysis based on our previous mRNA sequencing data to analyze the expression pattern between the two breeds (Figure 8B). The significant differences in the expression of miRNAs and their targets between the two periods suggested the possible biological role miRNAs might play in mammary gland development and lactation.

### 2.9. Validation of miRNA Expression with Quantitative RT-PCR

**Figure 9:**
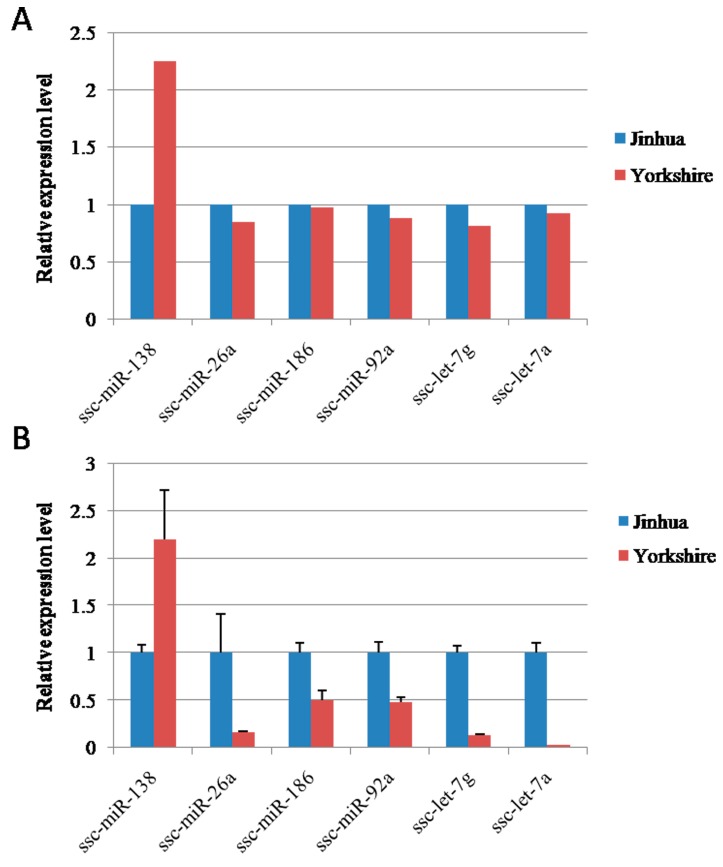
Verification of miRNA sequencing results by real-time PCR. (**A**) sequencing data and (**B**) qPCR data.

To validate the reliability of the sequencing data, we applied relative real-time quantitative RT-PCR (Figure 9B) to compare the expression levels of the differentially expressed miRNAs with sequencing results (Figure 9A). The expression levels of six differentially expressed miRNAs were selected randomly and validated in RX_J and RX_Y. In general, the expression pattern was consistent with the Solexa sequencing results.

## 3. Discussion

The mammary gland is important to all mammal species. In multiparous pigs, the number and the shape of functional mammary gland complexes are major determinants of the mothering ability of sows. Sow milk yield and quality is crucial for the survival and growth of piglets [16]. The mammary gland prepares for lactation by undergoing extensive growth and differentiation during pregnancy. The extent of this development determines sow lactation performance, which, in turn, regulates piglet growth rate. Indeed, milk production can limit piglet growth rate starting from day 8 to 10 of lactation, where the growth of piglets during lactation determines their subsequent growth rate during the finishing period [17,18].

MicroRNAs, as key components of most of the regulatory events, play important roles at the post-transcriptional level in various developmental and physiological processes. Some studies showed that miRNAs were involved in the regulation of the mammary gland, the organ that can undergo cycles of cell division, differentiation and dedifferentiation [19,20]. Despite increasing efforts in miRNAs identification across various species and diverse tissue types, little is known about porcine mammary gland miRNAs. All the published RNA expression studies conducted on porcine mammary gland up to this point have focused on different developmental stages or breast milk exosomes [21], and this is the first publication of miRNA sequencing analysis of porcine lactating mammary gland tissues. High-throughput massively parallel RNA sequencing is a recently developed approach and is rapidly emerging as a more powerful alternative platform to microarrays for whole-genome expression profiling. Such next generation sequencing technologies offer many potential advantages compared with microarrays [22].

In this study, miRNA expression profiles of mammary glands in two breeds of sows at day 21 of lactation were identified using miRNA sequencing technology. The Jinhua pig, named after Jinhua city in Zhejiang province of eastern China, is a traditional, slow-growing breed and is popular for its superior quality pork [23]. The Yorkshire pig is a world famous pig breed which represents the fast growing lean type [24]. The lactation performance between the two differs significantly, including milk composition and milk production. Yorkshire sows produce more milk while milk of Jinhua sows contain more energy content which can make the two different breeds good models to study the impact of miRNA on mammary gland development and peak lactation performance.

Firstly, an analysis was performed to verify the microstructure of the lactating mammary gland tissues used in the study. Mature alveolar structures, α-casein and Cytokeratin-18 were detected using HE staining, immunohistochemistry and immunofluorescence methods. Secondly, two small RNA libraries generated a total of 39 M sequencing reads, from which 37 M reads of mappable sequences were derived. In total, 406 known miRNAs, 135 conserved miRNAs and 2823 novel miRNAs were detected in Jinhua lactating mammary gland; 406 known miRNAs, 138 conserved miRNAs and 2286 novel miRNAs were detected in Yorkshire lactating mammary gland. The results also suggested that the two parallel libraries were highly correlated and can well represent the true expression pattern.

Other data analysis including length distribution, chromosomal locations and conservation analysis were all conducted for better characterization of miRNA isolated from porcine mammary gland tissue. The comparison of miRNA expression levels between Jinhua and Yorkshire showed that large amounts of miRNA were differentially expressed. Real-time qPCR was later performed to validate the sequencing results. GO and KEGG analysis demonstrated that these differentially expressed miRNAs were involved in many signaling pathways that may be related to mammary gland function, such as the mTOR signaling pathway [25,26]. Previous studies have identified several lactation-related miRNAs that positively or negatively regulate mammary gland cell development by interacting with target mRNAs. Tanaka reported that miR-101 controls mammary gland development by regulating cyclooxygenase-2 expression [27]. In our study, miR-126 expression level in Jinhua was higher than that in Yorkshire and Wei Cui reported miR-126-3p regulates progesterone receptors and involves development and lactation of mouse mammary gland [28].

Therefore, to investigate the relationship between miRNAs and lactation, related miRNAs were selected for further evaluation. Target-predicting softwares, Targetscan and Miranda, were used to identify putative targets and to investigate the possible roles of these differentially expressed miRNAs in the regulation of gene expression. Interestingly, according to our data analysis, many of these target genes were reported to be related to the proliferation and viability of mammary epithelial cells. Then five target genes, STAT3, DNMT1, PTEN, PCNA, WNT1, in the study were chosen to perform hierarchical cluster analysis based on our previous mRNA sequencing data (not published) to analyze the expression pattern between the two breeds.

STAT3 belongs to the signal transducer and activators of transcription (STAT) family of transcription factors. The inactivation of Stat5 and Stat3 has demonstrated unique roles in mammary physiology. Inactivation of the genes encoding Stat5a and 5b has revealed their essential role in the proliferation and differentiation of mammary alveolar epithelium [29]. DNMT1, which is a DNA methyltransferase enzyme, mediates the transfer of methyl groups from *S*-adenosylmethionine to the 5 position of cytosine bases in the dinucleotide sequence CpG. Studies have shown that DNMT1 is abnormally expressed in many tumor types. Furthermore, DNMT1 was found in breast cancer and verified as a target for miR-148 [30]. PTEN (phosphatase and tensin homolog deleted from chromosome 10) is the first phosphatase identified as a tumor suppressor. PTEN null mammary epithelial cells were dysregulated and hyperproliferative. Mutant females developed mammary tumors early in life suggesting that PTEN plays an essential and cell-autonomous role in controlling proliferation, differentiation and apoptosis of mammary epithelial cells [31]. Proliferating cell nuclear antigen (PCNA) protein is one of the central molecules responsible for decisions of life and death of the cell. When not engaged in DNA replication, PCNA commits cells to cell cycle arrest and repair of DNA damage, or, when repair is not possible, absence or low levels of functional PCNA may drive cells into apoptosis [32]. GH (growth hormone) induces the production of IGFs (insulin-like growth factor) in the liver, and IGF signaling is important for mammary gland development. Estrogens, progesterone, and prolactin can act sequentially on the mammary epithelium in synergy with corticosteroids to orchestrate mammary gland development in the presence of GH acting possibly via stromal and epithelial cell [33]. WNT (Wingless and INT-1) paracrine signaling molecules play key roles in the development of most organ systems, regulating cell fate decisions, proliferation, adhesion, cell shape and cell movements. Chu reported that WNT signaling promotes placode development and is required for initiation of mammary gland morphogenesis [34]. In general, all five genes are involved in cell growth, apoptosis or signal transduction and play important roles in mammary gland development. The observed miRNA-mRNA interaction provides important insights into global miRNA-mRNA relationships in the porcine mammary gland.

## 4. Experimental Section

### 4.1. Ethics Statement

Experiments were performed according to the Regulations for the Administration of Affairs Concerning Experimental Animals and approved by the Institutional Animal Care and Use Committee at Zhejiang University, Zhejiang, China. Animals were allowed access to food and water *ad libitum* under controlled environmental conditions and were humanely sacrificed as necessary to ameliorate suffering.

### 4.2. Sample Collection and RNA Extraction

The mammary gland samples were collected from three Jinhua pigs and Yorkshire pigs respectively at 21-day lactation. All samples were immediately frozen in liquid nitrogen and stored at −80 °C. Total RNA was extracted using Trizol reagent (Invitrogen, Carlsbad, CA, USA) following the manufacturer’s procedure. Total RNA quantity and purity were analyzed with the Bioanalyzer 2100 and RNA 6000 Nano LabChip Kit (Agilent, Palo Alto, CA, USA) with RIN number >7.0.

### 4.3. Small RNA Library Construction and Sequencing

Approximately 1 µg of total RNA was used to prepare a small RNA library according to the protocol of TruSeq Small RNA Sample Prep Kits (Illumina, San Diego, CA, USA). We performed the single-end sequencing (36 bp) on an Illumina Hiseq2500 at the LC-BIO (Hangzhou, China) following the vendors recommended protocol.

### 4.4. Data Processing

Briely, the raw reads were subjected to the Illumina pipeline filter (Solexa 0.3), and then the dataset was further processed with an in-house program, ACGT101-miR (LC Sciences, Houston, TX, USA) to remove adapter dimers, junk, low complexity, common RNA families (rRNA, tRNA, snRNA, snoRNA) and repeats. Subsequently, unique sequences with lengths of 18–26 nucleotide were mapped to specific species precursors in miRBase 20.0 by BLAST search to identify known miRNAs and novel 3p- and 5p-derived miRNAs. Length variation at both 3' and 5' ends and one mismatch inside of the sequence were allowed in the alignment. The unique sequences mapping to specific mature species miRNAs in hairpin arms were identified as known miRNAs. The unique sequences mapping to the other arm of known specific species precursor hairpin opposite to the annotated mature miRNA-containing arm were considered to be novel 5p- or 3p-derived miRNA candidates. The remaining sequences were mapped to other selected species precursors (with the exclusion of specific species) in miRBase 20.0 by BLAST search, and the mapped pre-miRNAs were further BLASTed against the specific species genomes to determine their genomic locations. The above two were defined as known miRNAs. The unmapped sequences were BLASTed against the specific genomes, and the hairpin RNA structures containing sequences were predicated from the flank 80 nt sequences using RNAfold software [35]. 

### 4.5. Analysis of Differential Expressed miRNAs

miRNA differential expression based on normalized deep-sequencing counts was analyzed by selectively using Fisher exact test, Chi-squared 2 × 2 test, Chi-squared nXn test, Student’s *t*-test, and ANOVA based on the experiments design. The significance threshold was set to be 0.01 and 0.05 in each test.

### 4.6. The Prediction of Target Genes of miRNAs

To predict the genes targeted by differentially expressed miRNAs, two computational target prediction algorithms (TargetScan 5.0 and miRanda 3.3a) were used to identify miRNA binding sites. Finally, the data predicted by both algorithms were combined and the overlaps were calculated. The gene ontology (GO) terms and KEGG Pathway of these differentially expressed miRNA targets were also annotated.

### 4.7. Histologic Examination

Blocks of mammary gland tissue were fixed in 4% formalin for 48 h, processed and embedded into paraffin blocks according to routine procedures. The paraffin-fixed blocks were serially sectioned into 8 μm coronal slices and stored at −20 °C until further use. For routine histological studies, paraffin sections were stained with hematoxylin and eosin (HE). HE-stained sections were analyzed by light microscopy using a Nikon fluorescence microscope (Nikon, Tokyo, Japan).

### 4.8. Immunohistochemical Analysis and Immunofluorescence Assay

Casein protein expression was performed by immunohistochemistry and CK-18 was detected in frozen sections by immunofluorescence method. Anti-casein and CK-18 primary antibodies were purchased from Abcam (Cambridge, UK). Immunohistochemistry analysis was performed according to general protocol steps. For immunofluorescence assay, sections were fixed with 4% formaldehyde for 10 min. The slides were then rinsed three times in PBS for 5 min each and blocked for 60 min. The blocking solution was replaced by primary antibody solution (1:100), and the samples were incubated overnight at 4 °C. The next day, slides were rinsed three times in PBS for 5 min each. CY3-conjugated secondary antibody (1:200, Jackson, Lancaster, PA, USA) with DAPI was added, and the slides were incubated for 1 h at 37 °C in the dark, followed by three rinses in PBS for 5 min each. The specimens were viewed under a fluorescence microscope (Nikon, Japan).

### 4.9. Quantitative RT-PCR Assay

The miRNA expression assay were analysed using real-time PCR. Total RNA were extracted from the mammary gland tissues in both breeds separately using Trizol reagent (Invitrogen, Calsbad, CA, USA). Real-time PCR was performed on an ABI Step One Plus system (Applied Biosystem, Calsbad, CA, USA) using SYBR Premix Ex Taq™ kit (TAKARA, Tokyo, Japan) with specific primers. All reactions were run in triplicate. Beta-actin was used as a gene assay control and met-rRNA as a miRNA control. Fold changes were determined by the threshold cycle (*C*_t_). Fold changes of miRNA expression were calculated using the 2^−ΔΔ*C*t^ method, where Δ*C*_t_ = (*C*_t_ target − *C*_t_ control) sample.

### 4.10. Statistical Analysis

All data were analyzed using SPSS software (V16.0, SPSS Inc., Chicago, IL, USA). Values in the texts and figures represent the results of at least three separate experiments. Group comparisons were performed using ANOVA with the Student’s *t*-test. Differences were considered statistically significant at *p* < 0.05.

## 5. Conclusions

In summary, our analysis revealed miRNA expression profiling in phenotypically divergent swine breeds. The main aim of this study was to identify miRNAs that may play regulatory roles in the porcine lactating mammary gland by comparing their expression patterns in Jinhua and Yorkshire swine. A large variety of miRNAs were found to be related to mammary gland development and lactation by interaction with putative target genes. These findings suggest that miRNA expression patterns may contribute significantly to target mRNA regulation and influence mammary gland development and peak lactation performance. We have demonstrated here that miRNA sequencing technology can be applied to provide a framework for understanding the relationship of miRNA regulation to changes in gene expression.

## Supplementary Materials

Supplementary materials can be found at http://www.mdpi.com/1422-0067/16/01/1448/s1.
